# Special Issue on “Terrestrial Laser Scanning”: Editors’ Notes

**DOI:** 10.3390/s19204569

**Published:** 2019-10-21

**Authors:** Joan R. Rosell-Polo, Eduard Gregorio, Jordi Llorens

**Affiliations:** 1 Research Group in AgroICT & Precision Agriculture, Department of Agricultural and Forest Engineering, Universitat de Lleida—AGROTECNIO Center, Av. Rovira Roure 191, 25198 Lleida, Spain; egregorio@eagrof.udl.cat (E.G.); jordi.llorens@eagrof.udl.cat (J.L.); 2 Research Group in AgroICT & Precision Agriculture, Department of Agricultural and Forest Engineering, Universitat de Lleida, Edifici CREA, c/Pere de Cabrera s/n, 25001 Lleida, Spain

**Keywords:** Terrestrial laser scanning, TLS, LiDAR, 3D modeling, point cloud, point cloud processing, stochastic models, sensor fusion, photogrammetry, structural diagnosis, deformation monitoring, change detection, structure from motion, diameter at breast height, understory structure, canopy characterization, olive orchards

## Abstract

In this editorial, we provide an overview of the content of the special issue on “Terrestrial Laser Scanning”. The aim of this Special Issue is to bring together innovative developments and applications of terrestrial laser scanning (TLS), understood in a broad sense. Thus, although most contributions mainly involve the use of laser-based systems, other alternative technologies that also allow for obtaining 3D point clouds for the measurement and the 3D characterization of terrestrial targets, such as photogrammetry, are also considered. The 15 published contributions are mainly focused on the applications of TLS to the following three topics: TLS performance and point cloud processing, applications to civil engineering, and applications to plant characterization.

## 1. Introduction

Since their availability a few decades ago, the applications of LiDAR sensors in 3D scanning have been growing steadily and have been extended to an increasing number of human activity areas and research fields. Specifically, an important effort has been devoted to the characterization of terrestrial targets, both through static and mobile LiDAR systems. The decrease in size and cost of many LiDAR sensors has led to their popularization among researchers and specialized service companies. At the same time, the emergence of autonomous vehicles research and development by many car-making companies has driven even more research into more advanced, miniaturized and cheaper LiDAR systems. Terrestrial laser scanning (TLS) use and applications have also benefited from the development of many, increasingly user-friendly software applications, either free or by payment, that offer a great number of functions and procedures to process the point clouds obtained by TLS measurements. Both measuring systems and processing software have also taken advantage of the increasing memory and processing capabilities of computer equipment. 

This special issue is devoted to the present innovative results, developments and applications in the area of terrestrial laser scanning (TLS), understood in a broad sense. Thus, although most contributions involved mainly use laser-based systems, other alternative technologies that also allow for obtaining 3D point clouds for the measurement and the 3D characterization of terrestrial targets, such as photogrammetry, have also been considered. 

## 2. Summary of the Special Issue

After the review phase, 15 out of 27 papers that were submitted to this special issue were accepted. These papers are mainly focused in the applications of TLS on the following three topics: TLS performance and point cloud processing, applications to civil engineering, and applications to plant characterization. In this section, the main objectives faced by each paper, as well as their results and conclusions are highlighted.

### 2.1. TLS Performance and Point Cloud Processing

In recent years, the growing availability of LiDAR systems has extended and has increasingly made the applications of TLS popular in a great variety of fields and applications. The most common output of TLS measurements are the so-called point clouds: a set of data corresponding to each sampled point of the measured object with its 3D coordinates information and, usually, some additional information about other variables, such as backscatter intensity, color of the object’s sampled point, and so on. The improving capabilities of both TLS and computers is leading to point clouds of increasingly large sizes, frequently having many gigabytes in file size and hundreds of millions of points. In this scenario, the development of automatic tools to process and extract the useful and desired features of the huge point clouds is a must.

In [1], a novel method for robust classification and segmentation of planar and linear features for the automatic extraction of building construction elements such as beams, slabs and columns, is proposed. The procedure begins with the classification of coplanar and collinear points with a principal components analysis, followed by their grouping through the new robust complete linkage clustering method. Independently of the previous stages, a robust method extracts the points of flat-slab floors and/or ceilings to improve computational efficiency. This method was evaluated in eight acquired datasets from a laboratory-environment and two construction sites. The resulting values of 96.8%, 97.7% and 95%, respectively, for precision, recall, and accuracy of the segmentation in the case of both construction sites demonstrate that the proposed method is suitable for robust segmentation of planar and linear features of even contaminated datasets, such as those typically collected from construction sites. An object’s edge detection is of particular interest in many practical situations, such as the characterization of plants and small objects, and has specific difficulties to be faced. For instance, in [2], the accuracy of a laser sensor in detecting the surface edge profiles of six different colored and textured complex-shaped objects including toy balls, rectangular cardboard boxes, basketballs, cylinders, and two different sized artificial plants, was evaluated. Three-dimensional images for each object’s surface at different LiDAR detection heights, travel speeds and horizontal distances were reconstructed. The comparison between the edge profiles of the six objects obtained with the laser system and the images taken with a digital camera was assessed using the edge similarity score (ESS), which, overall, averaged from 0.38 to 0.95 for all the objects under all the test conditions. EES was found to be mainly influenced by the horizontal distances of the objects, being the weaker influence when the objects were closer to each other, and to a lesser extent, by the other two variables. The authors of [3] present a novel algorithm, based on 3D LiDAR point clouds, for the automatic detection and extraction of the main characteristics of steel arches installed on a complex rock surface such as a tunnel. Firstly, a minimization of the density variance of the projected point cloud is proposed in order to calibrate the tunnel axis, followed by the segmentation of the rock surface from the tunnel point cloud. For this purpose, the region-growing method with the parameters obtained by the analysis of the tunnel section sequence is applied. Finally, the steel arches on the rock surface of the tunnel are detected with a directed edge growing (DEG) method. The results obtained in experiments undertaken in highway tunnels under construction show that the proposed algorithm can effectively extract the points of the edge of steel arches from 3D LiDAR point clouds of the tunnel without manual assistance, achieving 92.1% precision, 89.1% recall, and 90.6% F-score. 

Large 3D point clouds need proper methods to re-organize or index itself efficiently, such as octree, very popular for its speed, simplicity and memory efficiency. The authors of [4] introduce an efficient algorithm to build a file-based octree for a very large point cloud. To overcome the slowness of the initial proposed algorithm, especially for point clouds corresponding to rather long objects (this study was devoted to tunnels), a modification based on a semi-isometric octree group was implemented. After testing the method with point clouds measured in a long and a short tunnel and in an urban area, the authors proved that the proposed semi-isometric approach achieves a good balance between query performance and memory efficiency. In the case of medium sized point clouds and sufficient main computer memory, a memory-based approach is preferable, whilst a file-based semi-isometric approach is the better option when the point cloud is larger than the main computer memory. 

In tasks with high accuracy demand, including deformation analyses or precise volume measurements, such as those included in some papers of the present special issue, the performance of terrestrial laser scanners is an extremely important issue, being strongly dependent on the scanning conditions, such as the scanner design, configuration as well as the object surface geometry and reflectivity. The authors of [5] propose a method to determine the intensity-dependent range precision of 3D points for TLS by using range residuals in laser beam direction of a best plane fit, without requiring special targets or surfaces perpendicularly aligned to the scanner. This allows for an easier and quicker determination of the stochastic properties and model for the TLS measurements, which is very important information to get statistically reliable results. After investigating the different intensity types—raw and scaled—it is concluded that the intensity function can be obtained from raw intensity values, as written in literature or from scaled intensity values in the case of a restricted measurement volume, with no availability of the raw intensities. The authors of [6] analyze the range variance of a TLS as a least-squares power law function with respect to the intensity of the backscattered light. The authors investigate the effects of the variations of the intensity models’ parameters on the results of a least-squares fitting by means of closed loop simulations that keep the stochastic model and true geometry under control. After running simulations to a B-Spline approximation case study, it is concluded that, for an object having homogeneous properties, a constant variance can be assessed to all their points, without affecting the a posteriori variance factor or the loss of efficiency of the least-squares solution. These results are still valid when simulations are applied to more challenging geometries. In B-splines-based approximations, the study recommends dividing the TLS measured object into domains of homogeneous intensities to greatly simplify the computation of variance covariance matrix. 

Georeferencing of kinematic multi-sensor systems, greatly used to characterize 3D indoor and outdoor environments, is required in most cases for practical applications, i.e., both absolute position and orientation of the measuring platform should be continuously determined. There are many common environments where solutions for pose or position estimation, such as global navigation satellite systems (GNSS) and/or total station external tracking, are unreliable, inaccurate or simply not applicable. Based on a previous work that applied the iterated extended Kalman filter through implicit measurement equations and nonlinear equality constraints to georeference a simulated kinematic multi-sensor system, the authors of [7] extend it by nonlinear inequality state constraints. This increases the possibilities of applying any suitable geometrical pre-existing information and improves the resulting georeferencing even in difficult environments. An indoor kinematic multi-sensor system based on a TLS, with several combinations of state constraints and two types of inertial measurement units (IMU), is used to evaluate the approach presented. The results were obtained by combining real measurement data with simulated IMU measurements. In order to make the approach independent from the specific environment, the prior information and the multi-sensor system used, a more general overview of the filter approach is given. The authors conclude that the consideration of appropriate restrictions between the state parameters is desirable in terms of accuracy.

### 2.2. Applications to Civil Engineering

The characterization of terrestrial targets related to geosciences, architecture and industrial applications are among the fields where applications of TLS have recently experimented a higher outstanding growth. This is reflected in this special issue with about half of the published papers (eight) falling within this area. Three of these studies [1,3,4] have already been presented in the previous section because their objectives are mainly devoted to point cloud processing though focused and applied to civil engineering projects ([1] is applied to buildings and construction sites; [3] and [4] are applied to tunnels). Although it would also be appropriate, these three works will not be re-entered in this section. Remote sensing, and especially TLS, in civil engineering has recently been gaining attention in several specific areas, such as structural diagnostics, deformation assessment and so on, as these techniques allow for the creation of trustworthy three-dimensional models of the measured objects on a real scale and are relatively easy to use. Terrestrial laser scanners are fast and efficient, allowing to gather millions of points and building models that describe the 3D geometry of the objects of interest with high spatial resolution and accuracy.

The first two contributions of this section are devoted to the application of TLS-based measurements to pile testing. In [8], the authors investigate an alternative to the expensive and labor-intensive so-called inverted beam method, commonly used in static load tests of foundation piles. This method is subjected to systematic and difficult to detect errors coming from the displacement of the adopted reference beam to which the gauges are attached. In the framework of the application of geodetic methods, which allow for overcoming these drawbacks, in [8], an innovative instrumentation for a self-balanced stand for the static load test made from a closed-end, double steel pipe, is presented. The system is based on computer-controlled total station and TLS measurements instead of typical, precise geometric leveling. The assessment of the accuracy, as a result of the determination of the vertical displacements of both parts of the examined pile and their comparison with the results from the dial gauges, confirmed the usefulness of the developed measuring techniques. With the objective to appraise the morphology assessment of pile concrete surfaces in various geotechnical layers, the authors of [9] introduce an in situ research on the side surface of continuous flight auger foundation piles using a 3D TLS. After a two-step approach applied to six selected areas of each sample’s point cloud, seven height and four functional volume parameters according to the standard ISO 25178-2:2012 were determined. Results showed significant differences in the surface height and functional volume parameters’ values for each geotechnical layer where piles were formed. As the study of surface topography is key in the estimation of the shear strength at the pile–soil layer interface, the proposed methodology for pile morphology evaluation may be very valuable to improve the accuracy of the assumed friction factor between the concrete and soil as well as to improve the reliability of direct shear tests.

Two of the studies published deal with the application of TLS to the analysis of mechanical deformations. In this way, within the field of structural diagnostics, in [10], the authors face the challenge of improving point cloud processing by proposing a TLS-based general framework for studying the structural deformations of bridges based on advanced object shape analysis. The study also provides a comprehensive procedure for bridge evaluation, and adapts the spheres translation method for its use in bridge engineering. The method is concretized in a composite foot-bridge subjected to spatial deformations during the proof loading process. It is concluded that the proposed TLS-based method has several competitive advantages over the conventional total station and deflectometer-based methods: it is much faster and provides a better knowledge of the highly complex state of deformation, especially in bridges that are subject to very irregular deformation, such as composite bridges. In [11], the assessment of the evolution characteristics of the Yangshuli earth fissure hazard shows that the TLS technique can provide an effective solution in deformation monitoring of earth fissure hazards (a methodology that helps us to understand their formation mechanisms and to improve the effectiveness of planning and engineering actions). Time-series of high-density TLS-based point clouds through five sequential scans from 2014 to 2017 were used to detect the changes to ground surfaces and buildings in small and large scales, respectively. The analysis of local displacement revealed a tiny deformation on both the scarp and the walls, while global displacement analysis concluded that, even though the overall settlement slowly increases for the ground surface, the areas in the left side of scarp experiment show a relatively larger vertical displacement than those in the right side. 

The last article [12] of this section addresses the analysis of change detection of multitemporal point clouds datasets. Aiming to show a procedure to evaluate the morphological changes due to raw material extraction as well as its strengths and weaknesses, two topographic surveys, combining TLS with GNSS and Total Station studies, were carried out over two consecutive years in an underground marble quarry. The processing of multitemporal 3D point clouds allowed for the identification of the areas subjected to significant material extraction, the point clouds of which were converted into triangular meshes, allowing for the volume of the material extracted in one year of quarrying activities to be computed. The authors concluded that this case study demonstrates that TLS (and, by extension, structure from motion photogrammetry) allows for successful surveying and assessment of morphological changes in underground marble quarries through faster and more accurate data acquisition than classical survey measurements.

### 2.3. Applications to Plant Characterization

This last section includes three contributions of TLS measurements aiming to extract useful plant information, either individually or grouped. Two works focus on forestry canopies while the last is addressed to an agricultural crop. The authors of [13] present the accurate measurement of tree trunks or branches from dense point clouds obtained by employing a vertical optical flow with a fisheye camera. The images collected by the camera in a forest plot were used to create point clouds by means of photogrammetric-based commercial software, from which 3D models of the present tree trunks were obtained and mapped. To assess the accuracy of this image-based technique, the diameters of the trunks and their sections were compared, respectively, with the diameters measured manually at different heights and with the sections extracted from TLS-obtained point clouds. In this second case, the comparison of the cylindrical fitting of image-based and TLS-based point clouds gave great concordance, with average differences lower than 1 cm. For this application, the study concludes that the fisheye camera technique and TLS performances are comparable. TLS are key instruments to analyze spatial patterns of forests in detail. In order to examine the potential of TLS to fulfil objectives and efficiently characterize the complexity of understory forest structure, the authors of [14] present a new TLS-based quantitative approach, which was tested in an understory reference inventory site and in several forests and national parks with different management types. After suggesting the point cloud-based understory complexity index (UCI), the authors show that the proposed approach gives plausible results, permits to distinguish a variety of stand situations, and allows for their quantitative comparison. As a result, a better insight of the understory structures of forests, their complexity, drivers and dependents, can be achieved. 

Canopy characterization is gaining more and more attention when it comes to optimizing agricultural tasks. Many sensors and techniques give satisfactory results, among which, TLS has emerged as a key technology for 3D crop characterization and plant phenotyping. The study presented in [15] investigates a specific commercially available multi-beam 2D laser system for characterizing canopy trees in real-time conditions. Its accuracy at different distances and target’s density, the measurement cone width with different reflectivity targets and its ability to characterize both isolated and hedgerow olive orchards were tested in laboratory and field conditions. Laboratory results concluded that the cone width depends on the target reflectivity, the accuracy decreases with the target density and the measuring errors lay below 20 mm (1% relative error) except at 1.0 m distance, when relative error raised to 6%. As for the geometric estimations resulting from field tests, whilst the hedgerow tree volume was accurately estimated, the tree volumes of isolated olive trees were severely over-estimated, which could significantly limit its application in irregular canopy profiles. The authors conclude that the evaluated sensor offers an acceptable accuracy for several common agricultural operations, but the lower measuring errors of common LiDAR-based systems make them more suitable in case of the canopy volumes estimations.
